# Recommendation of the Advisory Committee on Immunization Practices for Use of a Third Dose of Mumps Virus–Containing Vaccine in Persons at Increased Risk for Mumps During an Outbreak

**DOI:** 10.15585/mmwr.mm6701a7

**Published:** 2018-01-12

**Authors:** Mona Marin, Mariel Marlow, Kelly L. Moore, Manisha Patel

**Affiliations:** ^1^Division of Viral Diseases, National Center for Immunization and Respiratory Diseases, CDC; ^2^Tennessee Immunization Program, Tennessee Department of Health, Nashville; ^3^Assistant Clinical Professor of Health Policy, Vanderbilt School of Medicine, Nashville, Tennessee.

A substantial increase in the number of mumps outbreaks and outbreak-associated cases has occurred in the United States since late 2015 (*1*,*2*). To address this public health problem, the Advisory Committee on Immunization Practices (ACIP) reviewed the available evidence and determined that a third dose of measles, mumps, rubella (MMR) vaccine is safe and effective at preventing mumps. During its October 2017 meeting, ACIP recommended a third dose of a mumps virus–containing vaccine* for persons previously vaccinated with 2 doses who are identified by public health authorities as being part of a group or population at increased risk for acquiring mumps because of an outbreak. The purpose of the recommendation is to improve protection of persons in outbreak settings against mumps disease and mumps-related complications. This recommendation supplements the existing ACIP recommendations for mumps vaccination (*3*).

In 1977, ACIP recommended 1 dose of mumps vaccine for all children aged ≥12 months (*4*). In response to multiple measles outbreaks in the late 1980s, in 1989 ACIP recommended routine administration of 2 doses of MMR vaccine for children, with the first dose administered at ages 12 through 15 months and the second at ages 4 through 6 years (*5*). In addition to improved measles control, this policy led to substantial reduction in the number of mumps cases in the United States during the 1990s, which was sustained through 2005 (*3*). However, in 2006, mumps outbreaks primarily affecting populations with high coverage with 2 doses of MMR vaccine in midwestern states and colleges resulted in 6,584 reported mumps cases that year (*6*). These outbreaks prompted ACIP to formally recommend a routine 2-dose mumps vaccination policy for school-aged children (i.e., kindergarten–grade 12) and adults at high risk (i.e., students at post-high school educational institutions, health care personnel, and international travelers) in 2006 (*7*). In addition, ACIP recommended that a second dose of mumps vaccine should be considered in outbreak settings for children aged 1–4 years and adults who have received 1 dose of vaccine, depending on the epidemiology of the outbreak (e.g., the age groups affected or institutions involved).

Despite this recommendation, mumps outbreaks continued to be reported throughout the United States, particularly in settings where persons have close, prolonged contact (e.g., universities and close-knit communities). To assist state and local health departments in responding to mumps outbreaks, CDC issued guidance on use of a third dose of MMR vaccine in the 2012 *Manual for the Surveillance of Vaccine-Preventable Diseases*.^†^ The guidance was based on limited data and provided criteria for health departments regarding when to consider use of a third dose in specifically identified target populations. Additional evidence on effectiveness and safety of the third dose of MMR vaccine recently became available and was presented to ACIP during 2017. This report summarizes the evidence considered by ACIP regarding use of a third dose of a mumps virus–containing vaccine during outbreaks and provides the recommendation for its use among persons who are at increased risk for acquiring mumps because of an outbreak.

## Methods

During March–October 2017, the ACIP Mumps Work Group held biweekly conference calls to review and discuss relevant scientific evidence. Topics addressed included the epidemiology of mumps in the United States since introduction of a routine second dose of MMR vaccine; effectiveness, duration of protection, immunogenicity, and risk factors for 2-dose vaccine failure; and effectiveness, immunogenicity, and safety of a third dose of MMR vaccine. Also assessed were stakeholders’ values attributed to the perceived benefits and harms of a third dose of MMR vaccine, acceptability, and implementation considerations regarding use of a third dose of MMR vaccine. Where scientific data were lacking, the summary of evidence incorporated the opinions of the Mumps Work Group member experts. Quality of evidence related to the benefits and harms of a third dose of mumps virus–containing vaccine was evaluated using the Grading of Recommendations, Assessment, Development and Evaluation (GRADE) framework (https://www.cdc.gov/vaccines/acip/recs/grade/about-grade.html). Methods and GRADE tables for the evidence for third dose of mumps virus–containing vaccine can be found at https://www.cdc.gov/vaccines/acip/recs/grade/mumps.html.

Summaries of the evidence reviewed were presented to ACIP at the February 2017, June 2017, and October 2017 meetings. At the October 2017 ACIP meeting, the proposed recommendation for a third dose of a mumps virus–containing vaccine (i.e., MMR or measles, mumps, rubella, and varicella [MMRV]) during mumps outbreaks was presented, and after a period for public comment, was approved unanimously by the voting ACIP members.^§^

## Summary of Key Findings

**Public Health Burden of Mumps.** Parotitis occurs in >85% of mumps cases; however, severe manifestations with complications such as orchitis (12%–66%), aseptic meningitis (0.2%–10%), or encephalitis (0.02%–0.3%) were recognized during the prevaccine era (*3*) and also can occur in vaccinated persons (3%–11%, <1%, and <0.3%, respectively) (*6*,*8*). Since 2012, the number of mumps cases, incidence, number of outbreaks, proportion of outbreak-associated cases, and number of jurisdictions reporting mumps outbreaks have all increased (*8*). The number of cases reported in 2016 (6,369) and 2017 (5,629, preliminary as of December 31) are the highest reported in a decade. Furthermore, from January 1, 2016 through June 30, 2017, state health departments reported 150 mumps outbreaks (the occurrence of three or more cases linked by place and time) (*9*), accounting for 9,200 cases; 39 (76%) of 51 of state health departments reported at least one outbreak (*2*,*8*). Seventy-five (50%) outbreaks occurred in universities and 16 (11%) in close-knit communities (i.e., communities or groups that are strongly connected by social, cultural, or family ties; participate in communal activities; or have a common living space). A median of 10 cases occurred per outbreak (interquartile range [IQR] = 4–26); 20 (13%) outbreaks had ≥50 cases, and these accounted for 83% of all outbreak-associated cases. Most cases occurred in young adults (median age of outbreak-associated patients = 21 years [IQR = 19–22]). Among 7,187 (78%) of 9,200 patients with known vaccination status, 5,015 (70%) had received 2 doses of MMR vaccine before developing mumps. The overall proportion of outbreak-associated mumps patients with complications was <3% (270 of 9,200); orchitis accounted for 75% (203 of 270) of reported complications. Other investigations also reported significantly lower prevalences of complications among mumps patients who had received 2 vaccine doses than among unvaccinated patients (*10*,*11*).

**Two-Dose Mumps Vaccine Effectiveness and Immune Response.** The median effectiveness of 2 doses of MMR vaccine in preventing mumps is 88%, with estimates ranging from 31% to 95% (*3*,*12*–*16*). The studies reporting these findings were conducted during 2005–2016, and most included persons who received the second MMR dose <10 years before the study. Several studies found decreasing effectiveness with increasing time after receipt of the second dose (*12*,*17*) or reported increased risk for mumps with increasing time after receipt of the second dose (*12*,*15*,*18*). Limited laboratory data on immune response to mumps virus indicate both lower antibody titers and poorer antibody quality (e.g., lower avidity antibodies, failure to generate strong memory B cell responses) after either natural mumps infection or mumps vaccination compared with the responses to infection with or vaccination against measles and rubella (*19*,*20*). Both neutralizing and non-neutralizing mean mumps antibody titers decline over time in persons who have received 2 doses of MMR vaccine (*19*,*21*–*23*).

Since 2006, the predominant circulating mumps virus genotype in the United States has been genotype G. Mumps virus–containing vaccines available in the United States are manufactured using the genotype A Jeryl-Lynn mumps virus strain (*3*). When studied 4–6 weeks and 10 years after receipt of the second MMR dose at age 4–6 years, all recipients had neutralizing antibody against genotype G mumps strain; however, the geometric mean titers of antibodies were lower than those against the vaccine strain (*21*,*24*).

**Third Dose of MMR Vaccine.** Three epidemiologic studies provided evidence regarding use of a third dose of MMR vaccine for prevention of mumps, all conducted in outbreak settings among populations with high coverage with 2 doses of MMR vaccine (schools and a university) (*12*,*25*,*26*). All studies reported lower attack rates among persons who received the third dose during the outbreak compared with persons who had received 2 doses before the outbreak, but only one study (*12*) found a statistically significant risk ratio (6.7 versus 14.5 per 1,000 person-years; p<0.001). Incremental vaccine effectiveness of the third versus the second MMR dose in these studies ranged from 61% to 88%, with one estimate being statistically significant (78.1%, 95% confidence interval = 60.9%–87.8%) (*12*). This study also found that students who had received 2 doses of MMR vaccine ≥13 years before the outbreak had nine or more times the risk for contracting mumps than did those who had received the second dose within the 2 years preceding the outbreak.

Two studies evaluated the geometric mean titers of mumps virus–specific antibodies after the third dose of MMR vaccine and demonstrated a significant increase (p<0.0001) 1 month after vaccination; however, antibody titers declined to near baseline by 1 year after vaccination (*27*,*28*). In the absence of a correlate of protection that would define the level of antibodies needed to protect a person from mumps disease, the clinical significance of these laboratory findings is unclear.

Five studies evaluated the safety of the third dose of MMR vaccine among children and young adults (aged 9–28 years) using passive and active surveillance for adverse events (J. Routh, CDC, personal communication, 2017) (*25*,*29*–*31*). No serious adverse events^¶^ were reported among 14,368 persons who received a third MMR vaccine dose. Nonserious adverse events were mild and reported at low rates. Among children, 6%–7% reported at least one nonserious adverse event within 2 weeks after receiving the third dose. Among young adults who received a third dose, the prevalences of four symptoms were significantly elevated during the 4-week postvaccination period compared with the prevaccination period. These symptoms and estimated proportions of subjects with episodes attributable to receipt of the third dose were lymphadenopathy (12%), diarrhea (9%), headache (7%), and joint pain (6%) (*32*). The median duration of these episodes was short (1–3 days).

**Stakeholders’ Values, Acceptability, and Implementation Considerations.** During July–September 2017, CDC conducted surveys of stakeholders, including students and parents, universities and colleges, and health departments to assess values, acceptability, and considerations for implementation of a third MMR vaccine dose during mumps outbreaks. Because the response rates for the student and parent surveys were very low (<0.5% in one university that agreed to participate), thereby limiting reliability of the results, the values regarding the benefits and harms of using a third dose to prevent mumps from the perspective of these stakeholders was based on expert opinion. Experts concluded that students and parents place high value on preventing mumps and its complications as well as preventing the harms associated with loss of productivity that can occur with mumps disease. Experts also concluded students and parents do not have concerns about safety of a third dose of MMR vaccine.

The survey of colleges and universities was distributed through the American College Health Association. Among 980 member university student health service administrators, 251 (26%) responded, representing colleges and universities from 47 states (*33*). Among these, 79 (31%) reported having mumps cases on campus since 2014. On a scale ranging from strongly negative (0), to neutral (5), to strongly positive (10), most university administrators felt student and parent attitudes were positive (80% and 83%, respectively, gave a score higher than 5 toward use of a third dose of MMR vaccine to protect students during a mumps outbreak (median = 7 for student attitudes, IQR = 6–9; median = 7 for parent attitudes, IQR = 6–8). With regard to disruption of activities, almost all administrator respondents indicated outbreaks resulted in some degree of disruption on campus. Using a scale from not disruptive (0), to somewhat disruptive (5), to extremely disruptive (10), 57% indicated that mumps outbreaks were more than somewhat disruptive (score >5) to student life (median = 6, IQR = 4–7), and 67% indicated outbreaks were more than somewhat disruptive to staff activities (median = 6, IQR = 5–8). Ranking of disruption to student life and staff activities did not differ significantly by the size of the outbreak experienced by the university (p = 0.20 and p = 0.57, respectively).

The survey of health departments was distributed through the Council of State and Territorial Epidemiologists to 81 health department jurisdictions, including 58 (72%) state and territorial health departments and 23 (28%) city or large urban health departments. Among the 61 (75%) responding health departments, 46 (75%) reported having one or more mumps outbreaks in their jurisdiction since January 1, 2016 (*33*). Nearly half (47%, 20 of 43) of health departments that reported outbreaks indicated recommending an outbreak dose or third dose of MMR vaccine** during one or more of these outbreaks. Compared with other mumps outbreak control measures, on a scale from not effective (0), to somewhat effective (5), to most effective (10), 42% (8 of 19) of health departments rated the intervention with an effectiveness score >5 (more than somewhat effective) (median = 5, IQR = 3–7). On a scale from least cost beneficial (0), to somewhat cost beneficial (5), to most cost beneficial (10), 53% (8 of 15) of health departments rated the intervention with a cost benefit score >5 (more than somewhat cost beneficial) (median = 7, IQR = 4–7).

**GRADE Quality of Evidence Summary.** The GRADE evidence type^††^ for critical outcomes was determined to be 4 for benefits (effectiveness for prevention of mumps) and 2 for harms (serious adverse events) (https://www.cdc.gov/vaccines/acip/recs/grade/mumps.html).

## Summary of Rationale for Recommendation for a Third Dose of Mumps Virus–Containing Vaccine in Persons at Increased Risk for Acquiring Mumps During an Outbreak

Mumps outbreaks have occurred primarily in populations in institutional settings with close contact or in close-knit communities. The current routine recommendation for 2 doses of MMR vaccine appears to be sufficient for mumps control in the general population, but insufficient for preventing mumps outbreaks in prolonged, close-contact settings, even where coverage with 2 doses of MMR vaccine is high. Waning of vaccine-induced immunity with time after receipt of the second vaccine dose in high intensity exposure settings typical of outbreaks contributes to this higher risk for mumps disease in these settings. Protection against severe disease, however, is maintained. Considering the evidence regarding the public health burden of disease and the known risk factors, persons who are at increased risk for acquiring mumps because of an outbreak were identified as a public health priority for receiving a third dose of mumps virus–containing vaccine.

A third dose of MMR vaccine has at least a short-term benefit for persons in outbreak settings. No serious adverse events were reported, and rates of nonserious adverse events were low. Because mumps is prevented in persons who receive a third dose, complications will also be prevented. Together, the benefit of added protection through administration of a third dose of MMR vaccine outweighs the low risk for vaccine-associated adverse events. Universities and health departments value the prevention of mumps disease and mumps complications and recognize that there is a potential loss of productivity because of mumps disease. A third dose of MMR vaccine was considered acceptable to students, parents, universities/schools, and health departments. Regarding implementation, an ACIP recommendation would allow health departments to make more rapid decisions regarding use of a third dose of MMR vaccine and increase access to vaccine for persons identified by public health authorities as being at increased risk for mumps because of an outbreak. MMRV vaccine, which is the other vaccine licensed in the United States for the prevention of mumps (*34*),^§§^ may also be used when a third dose mumps vaccination is indicated among children aged ≤12 years.

Available evidence indicates that a third dose of MMR vaccine improves protection for persons at increased risk for mumps because of an outbreak. Because of the complexity of mumps outbreaks, including the setting, the group or population affected, and risk factors for transmission, public health authorities are uniquely positioned to advise parents, students, clinicians, and universities regarding when and for which groups a third dose of MMR vaccine is appropriate. At this time, evidence is limited and is not sufficient to fully characterize the effect of a third dose of MMR vaccine on reducing the size or duration of an outbreak, nor are any data available to demonstrate the duration of additional protection conferred by a third dose. In addition, limited immunologic evidence suggests antibody titers decline within 1 year after the third dose. As more data on duration of protection after receipt of the third dose become available, evidence for use of a routine third dose will be considered. No evidence is available regarding the benefit of an additional dose of a mumps virus–containing vaccine to persons with documentation of receipt of 3 previous doses; therefore, no additional dose is recommended for persons in outbreak settings who have already received ≥3 doses of a mumps virus–containing vaccine.

## Recommendation

Persons previously vaccinated with 2 doses of a mumps virus–containing vaccine who are identified by public health authorities as being part of a group or population at increased risk for acquiring mumps because of an outbreak should receive a third dose of a mumps virus–containing vaccine to improve protection against mumps disease and related complications.

## Implementation Considerations and Future Research

In the setting of an identified mumps outbreak, public health authorities should define target groups at increased risk for mumps during the outbreak, determine whether vaccination of at-risk persons is indicated, and provide recommendations for vaccination to health care providers. Persons at increased risk for acquiring mumps are those who are more likely to have prolonged or intense exposure to droplets or saliva from a person infected with mumps, such as through close contact or sharing of drinks or utensils. During an outbreak, persons identified as being at increased risk and who have received ≤2 doses of mumps virus–containing vaccine or have unknown vaccination status should receive 1 dose. Additional guidance can be found in the *Manual for the Surveillance of Vaccine-Preventable Diseases* (*9*).

Contraindications and precautions for administration of a third dose of a mumps virus–containing vaccine are the same as those for routine use of the vaccine (1 or 2 doses) (*3*). CDC will monitor the burden of mumps among persons who have received 2 and 3 doses of mumps virus–containing vaccine and the duration of protection conferred by the third dose, as well as adverse events after the receipt of a third dose of a mumps virus–containing vaccine. Adverse events occurring after administration of any vaccine should be reported to the Vaccine Adverse Event Reporting System (VAERS; https://vaers.hhs.gov/). In addition, CDC will continue to collect data to assess the impact of receipt of a third dose of mumps virus–containing vaccine on mumps outbreaks.

## Footnotes

* The third dose may be administered as measles, mumps, rubella (MMR) vaccine, (M-M-R II, Merck & Co., Inc.) or measles, mumps, rubella, and varicella (MMRV) vaccine (ProQuad, Merck & Co., Inc.).

† This publication has been archived and is no longer available online. Readers may contact ncirddvdmmrhp@cdc.gov for more information.

§ Indication for a third dose of mumps virus–containing vaccine was not included in the package insert for these vaccines at the time the recommendation was made.

¶ Serious adverse events are defined as death, life-threatening illness, hospitalization or prolongation of existing hospitalization, or permanent disability.

** An outbreak dose is a dose of MMR vaccine administered without checking individual records before vaccination. Third dose of MMR vaccine is an MMR dose administered after confirmation of receipt of 2 MMR vaccine doses.

†† The evidence type (or quality of the body of evidence) is assessed for each outcome on the basis of the study design and specified downgrading or upgrading criteria. The evidence type is classified as the following: 1 = randomized controlled trials (RCTs), or overwhelming evidence from observational studies; 2 = RCTs with important limitations, or exceptionally strong evidence from observational studies; 3 = observational studies, or RCTs with notable limitations; 4 = clinical experience and observations, observational studies with important limitations, or RCTs with several major limitations.

§§ MMRV vaccine contains the same strain of mumps virus as MMR vaccine. MMRV vaccine was licensed on the basis of non-inferior immunogenicity compared with administration of MMR and varicella at the same time, therefore the two vaccination options are considered to provide the same protection against the respective diseases. MMRV vaccine is associated with an increased risk for fever and febrile seizures among children aged 12-23 months of age during the 5-12 days after the first dose compared with the use of MMR vaccine and varicella vaccine at the same visit. However, among children who received the second dose of MMRV vaccine at age 4-6 years data do not suggest an increased risk for febrile seizures.
